# Current situation of menstruation and gynecological diseases prevalence among Chinese women: a cross-sectional study

**DOI:** 10.1186/s12905-022-01860-5

**Published:** 2022-07-04

**Authors:** Francis Manyori Bigambo, Dandan Wang, Yuqing Zhang, Sabitina Mrisho Mzava, Rongrong Dai, Xu Wang

**Affiliations:** 1 School of Public Health, Nanjing Medical University, Nanjing, 211166 China; 2 Department of Endocrinology, Children’s Hospital of Nanjing Medical University, 72 Guangzhou Rd, Nanjing, 210008 China; 3 Department of Obstetrics and Gynecology, Women’s Hospital of Nanjing Medical University, Nanjing Maternity and Child Health Care Hospital, Nanjing, China; 4 Muhimbili National Hospital, P. O. Box 65000, Dar es Salaam, Tanzania; 5 Nanjing Pukou Central Hospital, PuKou Branch Hospital of Jiangsu Province Hospital, Nanjing, China

**Keywords:** Pubertal timing, Menstruation, Gynecological diseases, Dysmenorrhea, Polycystic ovary syndrome, Ovarian dysfunction, Endometriosis, Prevalence

## Abstract

**Background:**

Gynecological diseases have been taken attention and studied worldwide. Although, no recent studies have delineated the magnitude of gynecological diseases among Chinese women. This study aims to evaluate the current situation of menstruation and gynecological diseases prevalence among Chinese women.

**Methods:**

A cross-sectional study was conducted at a hospital affiliated with Nanjing medical university in Nanjing, China between September 2021 and February 2022. A sample size of 977 women aged 18–52 years participated in a face-to-face interview questionnaire. Logistic regression was performed to determine whether pubertal timing and menstrual characteristics were associated with gynecological diseases.

**Results:**

The most prevalent gynecological disease was dysmenorrhea (45.96%), followed by polycystic ovary syndrome, PCOS (19.04%), uterine fibroids (14.23%), spontaneous abortion (13.20%), trouble conceiving (12.59%), ovarian dysfunction (11.16%) and endometriosis (4.09%). In the adjusted model, heavy bleeding with large clots was associated with an increased risk of dysmenorrhea (odds ratio, OR = 5.01, 95% Confidence interval, CI 2.26, 11.10; *p *= 0.000), while history of precocious puberty diagnosis was associated with a reduced risk of dysmenorrhea (OR = 0.50, 95%CI: 0.26, 0.94; *p *= 0.031). Regular menstrual cycle in the past 12 months and regular menstrual periods were associated with decreased risk of PCOS (OR = 0.44, 95%CI 0.30, 0.65; *p *= 0.000) and (OR = 0.52, 95%CI 0.36, 0.74; *p *= 0.000), respectively. Histories of early thelarche, early menarche, and precocious puberty diagnosis were associated with increased risk of ovarian dysfunction (OR = 1.96, 95%CI 1.25, 3.08, *p *= 0.004), (OR = 2.26, 95%CI 1.24, 4.13; *p *= 0.008) and (OR = 2.79, 95%CI 1.36, 5.74; *p *= 0.005), respectively. Heavy bleeding and heavy bleeding with large clots were associated with endometriosis (OR = 4.92, 95%CI 1.50, 16.15, *p *= 0.009) and (OR = 5.67, 95%CI 1.42, 22.56; *p *= 0.014), respectively.

**Conclusions:**

The prevalence of gynecological diseases is increasing among Chinese women and pubertal timing and menstrual characteristics may be associated with some gynecological diseases, specifically dysmenorrhea, PCOS, ovarian dysfunction, and endometriosis.

**Supplementary Information:**

The online version contains supplementary material available at 10.1186/s12905-022-01860-5.

## Background

Gynecological diseases are disorders that affect the female reproductive system. Some of the common gynecological diseases include dysmenorrhea, endometriosis, uterine fibroids, polycystic ovary syndrome, and ovarian dysfunction. These diseases have raised social and public health concerns [1].

Dysmenorrhea is a lower abdominal pain or uterine cramps a few days before and /or during menstruation. It is classified as primary in the absence of pelvic pathology and secondary in the presence of pelvic pathology or identifiable medical condition [2]. The global prevalence of dysmenorrhea is ranged from 45 to 95%, while in China it has been estimated to be 41.7% [3]. Dysmenorrhea affects daily living, academic performance, work productivity, and quality of life. This is because dysmenorrhea is one of the causes of nonattendance from school or work [3]. The risk factors for dysmenorrhea include family history, null parity, and smoking [4]. Other factors include earlier menarche [4–6], irregular or long cycles, and heavy bleeding [5, 7].

Endometriosis is a condition whereby tissue resembling the lining of the uterus develops outsides the uterus, which in turn causes pain or infertility [8]. Globally, endometriosis affects approximately 190 million (10%) women of reproductive age [9]. In China, the majority of women delay endometriosis diagnosis by approximately 13 years [10]. Yamamoto et al. have reported the prevalence of endometriosis among Asian women to be 12.9% with a range of 4.2–21.0% [11]. The factors associated with endometriosis include low birth weight, early age at menarche, short menstrual cycle, low BMI, and low parity [12].

Uterine fibroids (uterine leiomyomas) are benign smooth muscle tumors of the uterus that affect women of childbearing age [13]. The world prevalence of uterine fibroids is estimated from 4.5 to 68.6%, although it may vary based on the kind of investigation, diagnosis, and ethnicity of the study population [14]. The common symptoms of uterine fibroids include heavy menstrual bleeding (which may lead to anemia), pain, abdominal distension, and urinary and gastrointestinal symptoms [15, 16]. The risk factors for uterine fibroids include being African or Asian women ethnicities [17], early menarche, late menopause, family history of fibroids, obesity, older age, and hypertension [18].

Polycystic ovary syndrome (PCOS) is a most common endocrine disorder and a highly prevalent disorder among women of childbearing age. Approximately, 5–20 % of women of childbearing age have been affected by PCOS worldwide [19], while in China the prevalence of PCOS is estimated to be 10.01% [20]. The main characteristics of PCOS include hyperandrogenism, anovulation, insulin resistance, hyperinsulinemia, menstrual dysfunction, and reproductive disorders [20]. Women with PCOS are likely to develop gestational diabetes, type 2 diabetes, endometrial cancer, and venous thromboembolism, cardiovascular and cerebrovascular diseases [19, 21]. The causes of PCOS are idiopathic, however, endocrine dysfunction, genetic traits, and environmental factors have been linked to PCOS [21]. In addition, other epidemiological studies have also related PCOS with early pubarche and thelarche [22] as well as menarche timing [23].

Ovarian dysfunction/ primary ovarian insufficiency (POI) is the condition whereby the ovary stops functioning before the age of 40 years [24]. The condition is marked by a dearth of ovarian sex hormone and reduction of ovarian follicles, which in turn lead to subfertility or infertility [25] and menopause [26]. The global prevalence of POI is estimated to be 3.7%, and countries with medium and low human development index were observed to have a higher prevalence [27]. The complications of POI are skeletal fragility, cognitive disorder, and cardiovascular events [26]. The causes of POI are idiopathic, however, genetic, environmental, infections, metabolic disorders, iatrogenic procedures, and autoimmune disorders have been reported to be the causative agents [25, 28, 29]. Weghofer et al. have reported that POI is associated with early age at menarche [30]. In addition, women with menstrual cycle irregularities should be monitored for POI [31].

However, menstruation and gynecological diseases have been taken attention and studied worldwide, no recent studies have delineated the magnitude of these gynecological diseases among Chinese women. Therefore, we conducted a cross-sectional study to evaluate the current situation of menstruation and disease prevalence among Chinese women. In addition, we assessed the association between pubertal timing and menstrual characteristics with gynecological diseases such as dysmenorrhea, trouble conceiving, spontaneous abortion, endometriosis, uterine fibroids, polycystic ovary syndrome, and ovarian dysfunction (primary ovarian insufficiency).

## Methods

### Study design, sampling, and setting

This is a cross-sectional study conducted at a hospital affiliated with Nanjing medical university in Nanjing, China between September 2021 and February 2022. In the present study, the sample size was calculated by using the formula:

$$ {\text{N}} = \frac{{{\text{P}}(1 - {\text{P}}){\text{Z}}^{2} }}{{{\text{E}}^{2} }} $$

where N represents sample size; Z represents standard deviation = 1.96; E represents margin of error = 3.05% (0.0305); P represents the prevalence of disease. We selected the prevalence of dysmenorrhea (41.7%) [3] to calculate the sample size because dysmenorrhea was the most prevalent gynecological disease. A confidence interval of 95% was employed and the power of the study was 90%. The minimum sample size obtained was 1004, but in our study, we enrolled 1005 participants.

The gynecology physicians recruited all the participants by a systematic random sampling approach using a unique identification number from the hospital record. The eligibility criteria were women aged ≥ 18 years, with no history of active smoking and drinking, and who were willing to participate in the study. Exclusion criteria were women with other underlying conditions, particularly chronic conditions such as cancer, thyroid conditions, or some other hormonal conditions or even taking chemical or traditional/ herbal or various supplements.

### Data collection method

A face-to-face interview questionnaire of 24 questions was conducted at the gynecological health clinic to evaluate the current situation of menstruation and disease prevalence among Chinese women. The questionnaire is comprised of three parts. The first part included questions about baseline characteristics of the study participants such as willingness to participate in a study, survey start time, date of birth of the participants, height, body weight, ethnicity (Han and others), education background (never went to school, primary school, junior high school, high school/technical school, college, undergraduate, postgraduate and above), and working hours (regular day shift, regular night shift, irregular night shift, regular shift, irregular shift, and not working at present). Height was measured via a stadiometer to the nearest 0.1 cm barefoot and body weight was measured via a beam balance scale to the nearest 0.1 kg barefoot and in light indoor clothing [32]. Both height and body weight were measured by the gynecology physicians. Body Mass Index (BMI) was computed by dividing weight and height square [weight (kg)/height (m)^2^], then it was categorized into underweight (BMI < 18.5), normal weight (BMI = 18.5–24.9), overweight (BMI = 25–29.9) and obesity (BMI ≥ 30) [33]. The second part included questions about pubertal timing and menstrual characteristics such as chronological age at breast development (thelarche), chronological age at first menarche, history of precocious puberty diagnosis, regular menstrual cycles in the past 12 months (without hormonal contraceptives), a regular menstrual period in the past 12 months, menstrual quantity in the past 12 months (amount of menstrual bleeding), menstrual flow length in the past 12 months. Age at thelarche was categorized into early thelarche (≤ 10 years), normal thelarche (11–13 years), and late thelarche (≥ 14 years); Age at menarche was categorized into early menarche (< 12 years), normal menarche (12–15 years) and late menarche (≥ 16 years); menstrual quantity was categorized into a little, moderate, heavy and heavy with large clots and; menstrual flow length was categorized into < 3 days, 3–7 days, and > 7 days. The third part included questions about the history of being diagnosed with gynecological diseases such as dysmenorrhea, trouble conceiving (infertility), spontaneous abortion, endometriosis, uterine fibroids, PCOS, and ovarian dysfunction/ POI. The Medical Ethics Committee of Nanjing Medical University approved this study, and informed consent was obtained from all participants.

### Statistical analysis

Stata software version 17.0 (College Station, TX, USA, copyright number: 301706381456) was used for data analysis. We used a histogram graph to assess the normality of the quantitative variables and found all quantitative variables were normally distributed (have a bell-shaped curve). In the descriptive statistics, frequency, percentage, mean and standard deviation were used to evaluate baseline characteristics, pubertal timing and menstrual characteristics, and the prevalence of the gynecological conditions. The results were presented by mean ± SD for continuous variables and frequency and percentage for categorical variables. We further used univariate and multivariate logistic regressions to explore the association between pubertal timing and menstrual characteristics and gynecological diseases. The results were presented as odds ratio (OR), 95% confidence interval (CI), and pseudo-R^2^. In the multivariate model, we adjusted the model by age, race, education, working hours, and BMI. The level of significance was set at a *p* < 0.05.

## Results

Among 1005 women who enrolled in the study, 28 participants were excluded because were under 18 years old. The mean age of the subjects was 31.72 ± 7.97 years with a range of 18–55 years. Of 977 participants involved in computing the baseline characteristics, 75.75% were between the age range of 21–39 years and 37 were above 18 years but did not want to disclose their age. The majority of the participants (97.95%) were from the Han race. The highest education level was postgraduate and above (57.01%). Most of the participants had regular day shifts (56.09%) and normal weight (71.34%) (Table1).

**Table 1:** Baseline characteristics of the study participants (N = 977)

	Mean ± SD or N (%)
Age (year)	31.72 ± 7.97
Range (year)^a^	18–55
Missing	37
Age group (year)	
18–20	46 (4.89)
21–29	371 (39.47)
30–39	341 (36.28)
≥ 40	182 (19.36)
Missing	37
Race	
Han	957 (97.95)
Others	20 (2.05)
Education	
Primary school	1 (0.10)
Junior high school	4 (0.41)
High/technical school	72 (7.37)
College	133 (13.61)
Undergraduate	210 (21.49)
Postgraduate and above	557 (57.01)
Working hours	
Regular day shift	548 (56.09)
Regular night shift	28 (2.87)
Irregular night shift	49 (5.02)
Regular shift	79 (8.09)
Irregular shift	71 (7.27)
Not working at present	202 (20.68)
Height (cm)	162.27 ± 4.96
Body weight (kg)	58.62 ± 9.13
BMI (kg/m^2^)	22.27 ± 3.44
BMI group (kg/m^2^)	
Underweight (BMI < 18.5)	107 (10.95)
Normal weight (BMI = 18.5–24.9)	697 (71.34)
Overweight (BMI = 25–29.9)	139 (14.23)
Obesity (BMI ≥ 30)	34 (3.48)

BMI, Body Mass Index; SD, standard deviation

^a^Minimum–Maximum. Missing is not included in the percentage

The mean age at thelarche was 11.24 ± 1.36 years with a range of 11–15 years, while women who had histories of early thelarche and late thelarche onset were 27.33% and 2.76% respectively. On the other hand, the mean age of menarche was 13.19 ± 1.44 years with a range of 9–17 years, while women who had histories of early and late menarche were 9.62% and 6.45% respectively. Although, only 5.42% of the participants were diagnosed to have precocious puberty (Table 2). The majority of women (60.70%) did not have regular menstrual cycles in the past 12 months, 34.49% did not have regular menstrual periods in the past 12 months, 10.75% had heavy bleeding, and 3.89% had heavy bleeding with large clots. The mean menstrual flow length was 5.90 ± 1.42 days with a range of 1–12 days. Most women had a normal menstrual flow length of 3–7 days (93.35%) (Table 2).

**Table 2:** Pubertal timing and menstrual characteristics (N = 977)

	Mean ± SD or N (%)
Age at thelarche (year)	11.24 ± 1.36
Range (year)^a^	11–15
Age groups at thelarche (year)	
Early thelarche (≤ 10)	267 (27.33)
Normal thelarche (11–13)	683 (69.91)
Late thelarche (≥ 14)	27 (2.76)
Age at menarche	13.19 ± 1.44
Range (year)^a^	9–17
Age group at menarche (year)	
Early menarche (< 12)	94 (9.62)
Normal menarche (12–15)	820 (83.93)
Late menarche (≥ 16)	63 (6.45)
Ever diagnosed with precocious puberty	
No	924 (94.58)
Yes	53 (5.42)
Regular menstrual cycle in the past 12 months	
No	593 (60.70)
Yes	384 (39.30)
Regular menstrual periods in the past 12 months	
No	337 (34.49)
Yes	640 (65.51)
Menstrual quantity in the past 12 months (amount of bleeding)	
Little	225 (23.03)
Moderate	609 (62.33)
Heavy	105 (10.75)
Heavy with large clots	38 (3.89)
Menstrual flow length in the past 12 months (days)	5.90 ± 1.42
Range	1–12
Menstrual flow length in the past 12 months (days)	
< 3	14 (1.43)
3–7	912 (93.35)
> 7	51 (5.22)

SD, standard deviation

^a^Minimum–Maximum

**Figure 1:**
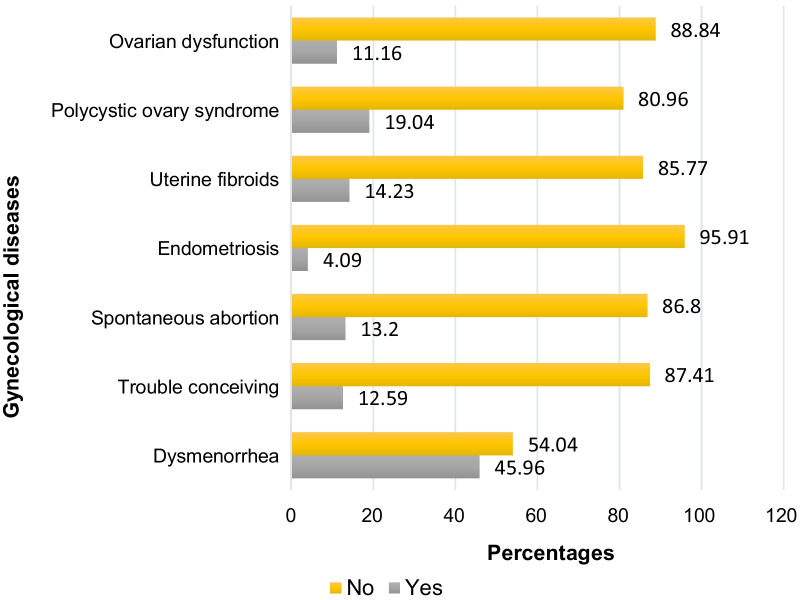
The prevalence of gynecological diseases among Chinese women (N = 977)

Figure 1 shows the prevalence of gynecological diseases among Chinese women (N = 977). Among gynecological diseases assessed in this study, dysmenorrhea in the past 12 months had the highest prevalence of 45.96%, followed by polycystic ovary syndrome (19.04%), uterine fibroids (14.23%), history of spontaneous abortion (13.20%), trouble conceiving (12.59%), ovarian dysfunction (11.16%), and endometriosis had the lowest prevalence (4.09%).

In the present study, women who had experienced precocious puberty were associated with decreased odds of dysmenorrhea (OR = 0.50, 95%CI 0.26, 0.94; *p *= 0.031), after adjusting for age, race, education, working hours, and BMI (Table 3). On the other hand, menstrual quantity whether moderate, heavy, or heavy with large clots was associated with increased odds of dysmenorrhea. Although in the adjusted model only heavy bleeding with large clots remained associated with increased odds of dysmenorrhea (OR = 5.01, 95%CI 2.26, 11.10; *p *= 0.000) compared with little bleeding.

**Table 3:** Association between pubertal timing and menstrual characteristics and dysmenorrhea (N = 940)

	Crude			Adjusted		
	OR (95%CI)	R^2*^	*P* value	OR (95%CI)	R^2*^	*P* value
Age groups at thelarche year)		0.000			0.047	
Normal thelarche (11–13)	Ref.			Ref.		
Early thelarche (≤10)	0.99(0.74, 1.32)		0.935	0.95(0.70, 1.28)		0.729
Late thelarche (≥ 14)	1.19(0.53, 2.68)		0.680	1.36 (0.57, 3.24)		0.490
Age group at menarche (year)		0.001			0.047	
Normal menarche (12–15)	Ref.			Ref.		
Early menarche (< 12)	0.93(0.60, 1.43)		0.740	0.95(0.60, 1.49)		0.817
Late menarche (≥ 16)	0.74(0.43, 1.27)		0.272	0.85(0.48, 1.52)		0.588
Ever diagnosed with precocious puberty		0.003			0.050	
No	Ref.			Ref.		
Yes	0.58(0.32, 1.05)		0.070	0.50(0.26, 0.94)		**0.031**
Regular menstrual cycle in the past 12 months	0.000			0.046		
No	Ref.			Ref.		
Yes	0.92(0.71, 1.19)		0.520	0.93(0.70, 1.23)		0.625
Regular menstrual periods in the past 12 months		0.001			0.047	
No	Ref.			Ref.		
Yes	1.19(0.91, 1.57)		0.200	1.18(0.88, 1.57)		0.267
Menstrual quantity in the past 12 months (amount of bleeding)		0.012			0.061	
Little	Ref.			Ref.		
Moderate	1.41(1.02, 1.93)		**0.036**	1.33(0.95, 1.86)		0.094
Heavy	1.67(1.03, 2.69)		**0.036**	1.63(0.99, 2.68)		0.053
Heavy with large clots	3.86(1.81, 8.23)		**0.000**	5.01(2.26,11.10)		**0.000**
Menstrual flow length in the past 12 months (days)		0.001			0.047	
< 3	Ref.			Ref.		
3–7	1.91(0.58, 6.26)		0.283	1.93(0.57, 6.54)		0.288
> 7	1.92(0.52,7.05)		0.328	1.94(0.51, 7.45)		0.333

Statistically significant values are given in bold

OR, odds ratio; CI, confidence interval; R^2*^, pseudo-R^2^; *p* < 0.05. N was after excluding the participants with missing values in age. Adjusted OR (95%CI) was obtained after adjusting for age, race, education, working hours, and BMI, Body Mass Index

No significant association was shown between pubertal timing and menstrual characteristics and trouble conceiving (Additional file 1: Table S1).

Women with late menarche had higher odds of spontaneous abortion (OR = 2.17, 95%CI 1.15, 4.09; *p *= 0.017), compared with those with normal menarche, and those with regular menstrual cycle in the past 12 months had higher odds of spontaneous abortion (OR = 1.46, 95%CI 1.00, 2.13; *p *= 0.048). On the other hand, women with moderate bleeding had lower odds of spontaneous abortion (OR = 0.65, 95%CI 0.42, 1.00; *p *= 0.048) compared with those with little bleeding. However, these associations were not observed in the adjusted model (Additional file 1: Table S2).

Women with late menarche had higher odds of endometriosis compared with those with normal menarche. Women with heavy bleeding (OR = 3.63, 95%CI 1.16, 11.39, *p *= 0.027), and heavy bleeding with large clots (OR = 6.59, 95%CI 1.81, 24.05; *p *= 0.004) were more likely to have endometriosis, compared with those with little bleeding. Although, in the adjusted model only women with heavy bleeding (OR = 4.92, 95%CI 1.50, 16.15, *p *= 0.009) and heavy bleeding with large clots (OR = 5.67, 95%CI 1.42, 22.56; *p *= 0.014) remained associated with higher odds of endometriosis compared with those with little bleeding (Table 4).

**Table 4:** Association between pubertal timing and menstrual characteristics and endometriosis (N = 940)

	Crude			Adjusted		
	OR (95%CI)	R^2*^	*P* value	OR (95%CI)	R^2*^	*P* value
Age group at thelarche (year)		0.003			0.098	
Normal thelarche (11–13)	Ref.			Ref.		
Early thelarche (≤10)	1.00(0.47, 2.11)		0.997	1.13(0.52, 2.45)		0.756
Late thelarche (≥ 14)	2.29(0.51, 10.27)		0.280	2.39(0.50, 11.54)		0.276
Age group at menarche (year)		0.018			0.106	
Normal menarche (12–15)	Ref.			Ref.		
Early menarche (< 12)	1.69(0.63, 4.50)		0.297	1.75(0.63, 4.90)		0.284
Late menarche (≥ 16)	3.32(1.31, 8.42)		**0.011**	2.59(0.93, 7.22)		0.068
Ever diagnosed with precocious puberty		0.000			0.094	
No	Ref.			Ref.		
Yes	1.00(0.23, 4.26)		0.996	1.00(0.22, 4.58)		0.996
Regular menstrual cycle in the past 12 months	0.000			0.094		
No	Ref.			Ref.		
Yes	1.06(0.54, 2.07)		0.870	0.95(0.47, 1.91)		0.889
Regular menstrual periods in the past 12 months		0.000			0.094	
No	Ref.			Ref.		
Yes	0.96(0.48, 1.91)		0.908	0.96(0.46, 1.96)		0.901
Menstrual quantity in the past 12 months (amount of bleeding)		0.037			0.130	
Little	Ref.			Ref.		
Moderate	1.41(0.52, 3.84)		0.496	1.65(0.59, 4.57)		0.338
Heavy	3.63(1.16, 11.39)		**0.027**	4.92(1.50, 16.15)		**0.009**
Heavy with large clots	6.59(1.81, 24.05)		**0.004**	5.67(1.42, 22.56)		**0.014**
Menstrual flow length in the past 12 months (days)		0.000			0.094	
< 3	Ref.			Ref.		
3–7	1.00(0.23, 4.27)		0.997	0.95(0.21, 4.29)		0.946
> 7	–			–		–

Statistically significant values are given in bold

OR, odds ratio; CI, confidence interval; R^2*^, pseudo-R^2^; *p* < 0.05. N was after excluding the participants with missing values in age. Adjusted OR (95%CI) was obtained after adjusting for age, race, education, working hours, and BMI, Body Mass Index.

“–”Indicate omitted data because of collinearity

Furthermore, women with late menarche (OR = 2.79, 95%CI 1.53, 5.09; *p *= 0.001) and heavy bleeding with large clots (OR = 2.35, 95%CI 1.06, 5.20; *p *= 0.036) were more likely to have uterine fibroids, compared with those with normal menarche and little bleeding, respectively. However, these associations were not present in the adjusted model (Additional file 1: Table S3).

Moreover, women with a regular menstrual cycle in the past 12 months (adjusted OR = 0.44, 95%CI 0.30, 0.65; *p *= 0.000) and regular menstrual periods in the past 12 months (adjusted OR = 0.52, 95%CI 0.36, 0.74; *p *= 0.000) had lower odds of the PCOS in both the crude and adjusted models (Table 5).

**Table 5:** Association between pubertal timing and menstrual characteristics and polycystic ovary syndrome (N = 940)

	Crude			Adjusted		
	OR(95%CI)	R^2*^	*P* value	OR (95%CI)	R^2*^	*P* value
Age group at thelarche (year)		0.006			0.095	
Normal thelarche (11–13)	Ref.			Ref.		
Early thelarche (≤ 10)	1.48(1.04, 2.11)		0.028	1.37(0.95, 1.99)		0.094
Late thelarche (≥ 14)	1.63(0.63, 4.20)		0.311	1.91(0.70, 5.26)		0.209
Age group at menarche (year)		0.002			0.091	
Normal menarche (12–15)	Ref.			Ref.		
Early menarche (< 12)	1.18(0.70, 2.00)		0.531	1.23(0.71, 2.15)		0.462
Late menarche (≥ 16)	0.67(0.31, 1.44)		0.302	1.02(0.45, 2.31)		0.964
Ever diagnosed with precocious puberty		0.003			0.094	
No	Ref.			Ref.		
Yes	1.67(0.88, 3.17)		0.114	1.86(0.93, 3.73)		0.079
Regular menstrual cycle in the past 12 months	0.026			0.110		
No	Ref.			Ref.		
Yes	0.41(0.28, 0.60)		**0.000**	0.44(0.30, 0.65)		**0.000**
Regular menstrual periods in the past 12 months		0.017			0.105	
No	Ref.			Ref.		
Yes	0.51(0.36, 0.71)		**0.000**	0.52(0.36, 0.74)		**0.000**
Menstrual quantity in the past 12 months (amount of bleeding)		0.002			0.093	
Little	Ref.			Ref.		
Moderate	0.89(0.60, 1.31)		0.548	0.82(0.54, 1.24)		0.338
Heavy	0.77(0.42, 1.42)		0.403	0.62(0.33, 1.19)		0.154
Heavy with large clots	0.59(0.22, 1.61)		0.306	0.59(0.20, 1.71)		0.330
Menstrual flow length in the past 12 months (Days)		0.000			0.091	
< 3	Ref.			Ref.		
3–7	0.77(0.21, 2.84)		0.697	0.71(0.18, 2.78)		0.626
> 7	0.83(0.19, 3.60)		0.807	0.71 (0.15, 3.28)		0.656

Statistically significant values are given in bold

OR, Odds ratio; CI, Confidence interval; R^2*^, pseudo-R^2*^; *p* < 0.05. N was after excluding the participants with missing values in age. Adjusted OR (95%CI) was obtained after adjusting for age, race, education, working hours, and BMI, Body Mass Index.

The results also showed that women with early thelarche (adjusted OR = 1.96, 95%CI 1.25, 3.08, *p *= 0.004) and early menarche (adjusted OR = 2.26, 95%CI 1.24, 4.13; *p *= 0.008) had higher odds of ovarian dysfunction compared with those with normal thelarche and normal menarche, respectively. Additionally, women with a history of precocious puberty diagnosis had higher odds of ovarian dysfunction (adjusted OR = 2.79, 95%CI 1.36, 5.74; *p *= 0.005). These associations were consistent in both the crude and adjusted models (Table 6).

**Table 6:** Association between pubertal timing and menstrual characteristics and ovarian dysfunction (N = 940)

	Crude			Adjusted		
	OR (95%CI)	R^2*^	*P* value	OR (95%CI)	R^2*^	*P* value
Age group at thelarche (year)		0.011			0.075	
Normal thelarche (11–13)	Ref.			Ref.		
Early thelarche (≤ 10)	1.78(1.16, 2.74)		**0.008**	1.96(1.25, 3.08)		**0.004**
Late thelarche (≥ 14)	1.41(0.41, 4.88)		0.583	1.27(0.34, 4.76)		0.724
Age group at menarche (year)		0.011			0.078	
Normal menarche (12–15)	Ref.			Ref.		
Early menarche (< 12)	2.13(1.21, 3.74)		**0.009**	2.26(1.24, 4.13)		**0.008**
Late menarche (≥ 16)	0.64(0.22, 1.80)		0.394	0.41(0.14, 1.23)		0.113
Ever diagnosed with precocious puberty		0.014			0.073	
No	Ref.			Ref.		
Yes	3.04(1.56, 5.91)		**0.001**	2.79(1.36, 5.74)		**0.005**
Regular menstrual cycle in the past 12 months		0.003			0.064	
No	Ref.			Ref.		
Yes	0.74(0.48, 1.14)		0.170	0.76(0.48, 1.21)		0.254
Regular menstrual periods in the past 12 months		0.006			0.066	
No	Ref.			Ref.		
Yes	0.67(0.44, 1.01)		0.056	0.71(0.46,1.09)		0.120
Menstrual quantity in the past 12 months (amount of bleeding)		0.003			0.069	
Little	Ref.			Ref.		
Moderate	0.86(0.53, 1.39)		0.532	0.78(0.47, 1.29)		0.324
Heavy	0.77(0.36, 1.66)		0.503	0.75(0.34, 1.67)		0.480
Heavy with large clots	0.40(0.09, 1.76)		0.225	0.25(0.05,1.14)		0.074
Menstrual flow length in the past 12 months (days)		0.003			0.066	
< 3	Ref.			Ref.		
3–7	0.69(0.15, 3.17)		0.635	0.60(0.12, 2.87)		0.519
> 7	0.35(0.05, 2.36)		0.282	0.26 (0.04, 1.86)		0.178

Statistically significant values are given in bold

OR, odds ratio; CI, confidence interval; R^2*^, pseudo-R^2*^; *p* < 0.05. N was after excluding the participants with missing values in age. Adjusted OR (95%CI) was obtained after adjusting for age, race, education, working hours, and BMI, Body Mass Index.

## Discussion

This study provides insight into the current situation of menstruation and diseases prevalence among Chinese women. It also assessed the association between pubertal timing and menstrual characteristics with gynecological diseases. To the best of our knowledge, our study is the first to evaluate simultaneously menstruation and diseases prevalence among Chinese women at the children’s hospital of Nanjing medical university.

Menarche is the hallmark of sexual maturity in females. It is usually developed in 2–3 years following thelarche onset. The median age for menarche has remained quite stable between 12 and 13 years in developed countries [34]. In the present study, the mean age at thelarche and menarche were 11.24 ± 1.36 years and 13.19 ± 1.44 years respectively. Women who had histories of early thelarche and early menarche development were 27.3% and 9.6% respectively, and those with histories of late thelarche and late menarche were 2.8% and 6.5%, respectively. Ansong et.al has documented that women who develop menarche later are likely to get menstrual irregularities [35].

In the current study, we found that 60.7 % of women had irregular menstrual cycles in the past 12 months, 34.5% had irregular menstrual periods in the past 12 months, 14.64% had heavy bleeding, and 89.2% had menstrual flow length of 3–7days. It has been estimated that almost 80% of women with normal ovulation have menstrual cycle intervals of 26–35 days, an average blood loss of 33.2 ml in a range of 10–84 ml, and menstrual flow lengths of 3–6 days in a range of 2–12 days [35].

Among gynecological diseases assessed in this study, the most prevalent was dysmenorrhea (45.96%), which is even higher than 41.7% reported in the previous study [3], indicating that dysmenorrhea is increasing among Chinese women. However, it is lower than that reported in other countries such as Ghana 68.1% [36], Ethiopia 71.69% [37], and Greece 89.2% [38]. Moreover, studies have observed that irregular or long cycles, and heavy bleeding were associated with an increased risk of dysmenorrhea [5, 7]. In the current study, only heavy bleeding with large clots was associated with an increased risk of dysmenorrhea. In addition, women who had a history of precocious puberty onset were less likely to get dysmenorrhea the mechanism underlying this act could not be elucidated. However, we can speculate that precocious puberty, specifically early menarche marks an earlier beginning of reproductive life and reduces the sensitivity of the uterus to prostaglandins. On the other hand, delayed puberty procrastinate reproductive life and increases the sensitivity of uterus to prostaglandins, which in turn causes severe menstrual pain [39]. In contrary, several studies have reported that early menarche is associated with increased dysmenorrhea [4–6]. This discrepancy should be further explored since another study has documented that pubertal timing, particularly age at menarche cannot determine the presence or absence of dysmenorrhea as pubertal timing and menstrual characteristics act independently [40].

A systematic review by Deswal et al. has reported that the global prevalence of PCOS is 21.27% [41]. A recent meta-analysis that pooled studies from China has reported the total prevalence of PCOS among Chinese women to be 10.01% [20], which is lower than that found in our study (19.04%). In India, PCOS is reported to be within a range of 3.7–22.5 % [42]. However, the prevalence of PCOS may differ based on the diagnostic criteria used, geographical location, and the study population [20, 41, 42], our findings indicate that PCOS is increasing among Chinese women. Moreover, we observed that women with regular menstrual cycles or periods in the past 12 months were less likely to get PCOS. This is because an irregular menstrual cycle is the authentication mark of PCOS [43, 44]. In addition, we did not find an association between pubertal timing and PCOS contrary to the previous studies [22, 23].

In this study, the prevalence of uterine fibroids was higher (14.23%) than that of 4.10% observed in 2016 in Chinese women [45]. These results indicate that the prevalence of uterine fibroids is increasing among Chinese women. Although, it is lower than that observed in the Middle East population, which is 30.8% within a range of 18.5–42.6% [44, 46], and in African women ethnicity approximately to be 70–80% [47]. Moreover, one study has reported the association between early menarche and uterine fibroids [18]. In the present study, there was not enough evidence to show the association between pubertal timing and menstrual characteristics, and uterine fibroids.

Spontaneous abortion is the loss of pregnancy innately prior to 20 weeks of gestation [48]. It is among the major complication of pregnancy [49]. A cross-sectional study of Chinese populations has reported the prevalence of spontaneous abortion to be 6.7% [50], which is less than that found in our study (13.20%). On the other hand, trouble conceiving (infertility), which is an inability to get pregnant following 12 months of unprotected sexual intercourse, is a worldwide reproductive health problem increasing in the prevalence by 0.37 % each year [51]. In China, a study conducted in Henan province among women aged 20–49 years has reported the prevalence of infertility to be 24.6% [52]. Another population-based cross-sectional study involving eight provinces in China has reported a prevalence of 25% [53]. Interestingly, in our study, the prevalence of trouble conceiving was 12.5%, indicating that the infertility rate is decreasing. However, this result should be interpreted with caution because the huge discrepancy between the previous studies and ours may be caused by different study designs, times, and regions as infertility rates vary with region and time [52]. In agreement with our study, a study conducted in Turkey from 1993 to 2003 showed a decrease in the infertility rate from 12 to 8.6% [54]. Furthermore, in a previous study, menstrual cycle characteristics were associated with spontaneous abortion and trouble conceiving [55]. However, in this study, there was not enough evidence to show the association between pubertal timing and menstrual characteristics with either trouble conceiving or spontaneous abortion. The associations observed in the unadjusted models were not consistent in the adjusted models. Importantly, these results revealed that the associations seen in the unadjusted models could be caused by the presence of unmeasured confounders.

In the current study, the prevalence of ovarian dysfunction among Chinese women was higher (11.16%) than the global prevalence (3.7%) reported in the previous study [27]. Previous studies have reported the risk factors for ovarian dysfunction to be early age at menarche [30] and menstrual cycle irregularities [31]. These results may be quite similar to our findings as women who had histories of early thelarche, early menarche, and precocious puberty diagnosis were more likely to develop ovarian dysfunction.

In our study, the prevalence of endometriosis was lower (4.19%) than other gynecological diseases evaluated among Chinese women. It was also lower than the world prevalence (10%) of endometriosis among women of childbearing age, lower than 9.5% among Asian women [11], and lower than 12. 9% among Middle East women [46]. A preceding study has reported that early age at menarche and short menstrual cycle were associated with endometriosis [12]. These associations, however, were not observed in the present study, instead in this study both heavy bleeding and heavy bleeding with large clots were associated with an increased risk of endometriosis among Chinese women. Although, there were wider confidence intervals in the results from both the crude and adjusted models (Table 4), indicating that this association could be due to the presence of uncontrolled confounding caused by unmeasured confounders. Since women with endometriosis are generally present with heavy bleeding [56].

The strengths of this study include we simultaneously assessed the prevalence of gynecological diseases among Chinese women and identify whether pubertal timing and menstrual characteristics were associated factors. Also, the sample size used was enough to ensure the reliability of our study. The limitations of this study include firstly, our study was a cross-sectional study in this type of study it is not possible to assess causality. Secondly, most of the information including pubertal timing and menstrual characteristics, and gynecological diseases was assessed through a questionnaire, which could lead to recall bias. Thirdly, our findings may be subjected to confounding because important variables such as socioeconomic status, physical activity, daily energy, and food intake were not measured in the current study for various reasons such as very low response rate or we could not quantify some index (physical activity). Additionally, there is a potential for residual confounding because most of the covariates were categorically controlled in the adjusted model. Given that, interpretation of our findings may be limited. Fourthly, the results found in this study cannot be generalized to all Chinese women. A population-based nationwide study is required to substantiate these findings and generalize the results to all Chinese women. Finally, regardless of such limitations pointed out, this study significantly revealed the prevalence of gynecological diseases among Chinese women, and pubertal timing and menstrual characteristics were related factors.

## Conclusions

This study found that the prevalence of gynecological diseases is increasing among Chinese women and pubertal timing and menstrual characteristics may be associated with some gynecological diseases, specifically dysmenorrhea, PCOS, ovarian dysfunction, and endometriosis. Our results suggest a need to increase awareness, effective prevention including early diagnosis, and proper management of these gynecological diseases to improve women's reproductive health and their quality of life. Also, a population-based nationwide study is needed to evident our findings and generalize the results.

## Supplementary Information

**Additional file 1. Table S1**. Association between pubertal timing and menstrual characteristics and trouble conceiving (N = 940). **Table S2**. Association between pubertal timing and menstrual characteristics and spontaneous abortion (N = 940). **Table S3**. Association between pubertal timing and menstrual characteristics and uterine fibroids (N = 940).

## Data Availability

The datasets used and/or analyzed during the current study are available from the corresponding author on reasonable request.

## Abbreviations

ACOG: American College of Obstetricians and Gynecologists
BMI: Body Mass Index
CI: Confidence interval
OR: Odds ratio
PCOS: Polycystic ovary syndrome
POI: Primary ovary insufficiency
SD: Standard deviation
TX: Texas
USA: United State of America
WHO: World Health Organization
