# Supplementary material for: Current situation of menstruation and gynecological diseases prevalence among Chinese women: a cross-sectional study
Source: BMC Womens Health. 2022 Jul 4;22:270. doi: 10.1186/s12905-022-01860-5 (PMC9254498; doi:10.1186/s12905-022-01860-5)
Supplement: Supplementary file 1 — Additional file 1. Table S1. Association between pubertal timing and menstrual characteristics and trouble conceiving (N = 940). Table S2. Association between pubertal timing and menstrual characteristics and spontaneous abortion (N = 940). Table S3. Association between pubertal timing and menstrual characteristics and uterine fibroids (N = 940). [file 12905_2022_1860_MOESM1_ESM.docx]

**Supplementary Material**

**Supplementary Tables**

| **Table S1:** Association between pubertal timing and menstrual characteristics and trouble conceiving (N=940) | | | | | | | |
| --- | --- | --- | --- | --- | --- | --- | --- |
|  | **Crude** | | |  | **Adjusted** | | |
|  | **OR (95%CI)** | R^2*^ | **P-Value** |  | **OR (95%CI)** | **R^2*^** | **P-value** |
| **Age groups at thelarche (Year)** |  | 0.004 |  |  |  | 0.030 |  |
| Normal thelarche (11-13) | Ref. |  |  |  | Ref. |  |  |
| Early thelarche (≤10) | 0.90(0 .58, 1.41) |  | 0.650 |  | 0.89 (0.56, 1.41) |  | 0.624 |
| Late thelarche (≥14) | 2.33 (0.90, 6.03) |  | 0.080 |  | 2.30(0.86, 6.17) |  | 0.099 |
| **Age group at menarche (Year)** |  | 0.001 |  |  |  | 0.026 |  |
| Normal menarche (12-15) | Ref. |  |  |  | Ref. |  |  |
| Early menarche (<12) | 1.28(0.70, 2.35) |  | 0.425 |  | 1.18(0.63, 2.20) |  | 0.607 |
| Late menarche (≥16) | 0.96(0.42, 2.17) |  | 0.923 |  | 1.06(0.45, 2.48) |  | 0.891 |
| **Ever diagnosed with precocious puberty** |  | 0.001 |  |  |  | 0.026 |  |
| No | Ref. |  |  |  | Ref. |  |  |
| Yes | 0.75(0.29 ,1.92) |  | 0.544 |  | 0.75(0.28, 1.99) |  | 0.566 |
| **Regular menstrual periods in the past 12 months** |  | 0.000 |  |  |  | 0.026 |  |
| No | Ref. |  |  |  | Ref. |  |  |
| Yes | 1.07(0.72, 1.59) |  | 0.735 |  | 1.01(0.67, 1.53) |  | 0.946 |
| **Regular menstrual periods in the past 12 months** |  | 0.001 |  |  |  | 0.027 |  |
| No | Ref. |  |  |  | Ref. |  |  |
| Yes | 0.86(0.58, 1.28) |  | 0.458 |  | 0.81(0.53, 1.23) |  | 0.321 |
| **Menstrual quantity in the past 12 months** |  | 0.003 |  |  |  | 0.029 |  |
| Little | Ref. |  |  |  | Ref. |  |  |
| Moderate | 0.84(0.53, 1.32) |  | 0.439 |  | 0.81(0.50, 1.29) |  | 0.368 |
| Heavy | 0.58(0.27, 1.28) |  | 0.178 |  | 0.61(0.27, 1.34) |  | 0.217 |
| Heavy with large clots | 1.16(0.45, 3.00) |  | 0.767 |  | 1.22(0.46, 3.27) |  | 0.927 |
| **Menstrual flow length in the past 12 months (Days)** |  | 0.001 |  |  |  | 0.027 |  |
| <3 | Ref. |  |  |  | Ref. |  |  |
| 3-7 | 0.77(0.17, 3.53) |  | 0.739 |  | 0.84(0.17, 4.04) |  | 0.829 |
| >7 | 1.05(0.19, 5.65) |  | 0.957 |  | 1.22(0.21, 6.96) |  | 0.824 |

Abbreviations: OR, Odds ratio; CI, Confidence interval; R^2*^, pseudo-R2; p-value <0.05. N was after excluding the participants with missing values in age. Adjusted OR (95%CI) was obtained after adjusting for age, race, education, working hours, and BMI (body mass index).

| **Table S2:** Association between pubertal timing and menstrual characteristics and spontaneous abortion (N=940) | | | | | | | |
| --- | --- | --- | --- | --- | --- | --- | --- |
|  | **Crude** | | |  | **Adjusted** | | |
|  | **OR (95%CI** | **R^2*^** | **P-value** |  | **OR (95%CI)** | **R^2*^** | **P-value** |
| **Age group at thelarche (Year)** |  | 0.004 |  |  |  | 0.089 |  |
| Normal thelarche (11-13) | Ref. |  |  |  | Ref. |  |  |
| Early thelarche (≤10) | 0.75(0.48, 1.17) |  | 0.206 |  | 0.85(0.53, 1.36) |  | 0.499 |
| Late thelarche (≥14) | 1.59(0.58, 4.36) |  | 0.369 |  | 1.90(0.64, 5.66) |  | 0.247 |
| **Age group at menarche (Year)** |  | 0.007 |  |  |  | 0.091 |  |
| Normal menarche (12-15) | Ref. |  |  |  | Ref. |  |  |
| Early menarche (<12) | 1.25(0.68, 2.29) |  | 0.469 |  | 1.17(0.61, 2.21) |  | 0.637 |
| Late menarche (≥16) | 2.17(1.15, 4.09) |  | **0.017** |  | 1.88(0.94, 3.76) |  | 0.074 |
| **Ever diagnosed with precocious puberty** |  | 0.001 |  |  |  | 0.087 |  |
| No | Ref. |  |  |  | Ref. |  |  |
| Yes | 1.40(0.66, 2.95) |  | 0.376 |  | 1.33(0.60, 2.95) |  | 0.483 |
| **Regular menstrual cycle in**  **the past 12 months** | | 0.005 |  |  |  | 0.090 |  |
| No | Ref. |  |  |  | Ref. |  |  |
| Yes | 1.46(1.00, 2.13) |  | **0.048** |  | 1.40(0.94, 2.10) |  | 0.098 |
| **Regular menstrual periods in the past 12 months** |  | 0.002 |  |  |  | 0.088 |  |
| No | Ref. |  |  |  | Ref. |  |  |
| Yes | 0.80(0.55, 1.18) |  | 0.270 |  | 0.83(0.55, 1.25) |  | 0.374 |
| **Menstrual quantity in the past 12 months (amount of bleeding)** |  | 0.010 |  |  |  | 0.091 |  |
| Little | Ref. |  |  |  | Ref. |  |  |
| Moderate | 0.65(0.42, 1.00) |  | **0.048** |  | 0.76(0.48, 1.21) |  | 0.248 |
| Heavy | 0.65(0.32, 1.31) |  | 0.231 |  | 0.77(0.37, 1.61) |  | 0.494 |
| Heavy with large clots | 1.56(0.68, 3.57) |  | 0.297 |  | 1.44(0.59, 3.53) |  | 0.425 |
| **Menstrual flow length in the past 12 months (Days)** |  | 0.001 |  |  |  | 0.088 |  |
| <3 | Ref. |  |  |  | Ref. |  |  |
| 3-7 | 0.52(0.14, 1.91) |  | 0.323 |  | 0.57(0.14, 2.25) |  | 0.419 |
| >7 | 0.45(0.10, 2.13) |  | 0.318 |  | 0.49(0.10, 2.55) |  | 0.400 |

Abbreviations: OR, Odds ratio; CI, Confidence interval; R^2*^, pseudo-R^2^; p-value <0.05. N was after excluding the participants with missing values in age. Adjusted OR (95%CI) was obtained after adjusting for age, race, education, working hours, and BMI (body mass index).

| **Table S3:** Association between pubertal timing and menstrual characteristics and uterine fibroids (N=940) | | | | | | | |
| --- | --- | --- | --- | --- | --- | --- | --- |
|  | **Crude** | | |  | **Adjusted** | | |
|  | **OR (95%CI)** | **R^2*^** | **P-value** |  | **OR(95%CI)** | **R^2*^** | **P-value** |
| **Age group at thelarche (Year)** |  | 0.000 |  |  |  | 0.261 |  |
| Normal thelarche (11-13) | Ref. |  |  |  | Ref. |  |  |
| Early thelarche (≤10) | 1.06(0.71, 1.60) |  | 0.770 |  | 0.72(0.44, 1.18) |  | 0.189 |
| Late thelarche (≥14) | 1.25(0.42, 3.75) |  | 0.686 |  | 1.18(0.32, 4.32) |  | 0.807 |
| **Age group at menarche (Year)** |  | 0.013 |  |  |  | 0.263 |  |
| Normal menarche (12-15) | Ref. |  |  |  | Ref. |  |  |
| Early menarche (<12) | 1.34(0.74, 2.43) |  | 0.330 |  | 0.62(0.31, 1.25) |  | 0.182 |
| Late menarche (≥16) | 2.79(1.53, 5.09) |  | **0.001** |  | 1.10(0.42, 2.90) |  | 0.849 |
| **Ever diagnosed with precocious puberty** |  | 0.000 |  |  |  | 0.258 |  |
| No | Ref. |  |  |  | Ref. |  |  |
| Yes | 1.15(0.53, 2.50) |  | 0.728 |  | 1.15(0.46, 2.91) |  | 0.762 |
| **Regular menstrual cycle in**  **the past 12 months** | | 0.000 |  |  |  | 0.258 |  |
| No | Ref. |  |  |  | Ref. |  |  |
| Yes | 1.08(0.74, 1.58) |  | 0.675 |  | 1.09(0.70, 1.69) |  | 0.719 |
| **Regular menstrual periods in the past 12 months** |  | 0.004 |  |  |  | 0.260 |  |
| No | Ref. |  |  |  | Ref. |  |  |
| Yes | 0.72(0.49, 1.04) |  | 0.083 |  | 0.76(0.49, 1.20) |  | 0.240 |
| **Menstrual quantity in the past 12 months (amount of bleeding)** |  | 0.010 |  |  |  | 0.260 |  |
| Little | Ref. |  |  |  | Ref. |  |  |
| Moderate | 0.80(0.51, 1.25) |  | 0.328 |  | 0.87(0.52, 1.46) |  | 0.602 |
| Heavy | 0.89(0 .45, 1.75) |  | 0.741 |  | 1.05(0.48, 2.31) |  | 0.905 |
| Heavy with large clots | 2.35(1.06, 5.20) |  | **0.036** |  | 1.39(0.53, 3.67) |  | 0.501 |
| **Menstrual flow length in the past 12 months (Days)** |  | 0.002 |  |  |  | 0.260 |  |
| <3 | Ref. |  |  |  | Ref. |  |  |
| 3-7 | 2.01(0.26, 15.62) |  | 0.503 |  | 1.64(0.17, 16.02) |  | 0.670 |
| >7 | 1.33(0.14, 12.52) |  | 0.801 |  | 0.94 (.077, 11.45) |  | 0.960 |

Abbreviations: OR, Odds ratio; CI, Confidence interval; R^2*^, pseudo-R^2*^; p-value <0.05. N was after excluding the participants with missing values in age. Adjusted OR (95%CI) was obtained after adjusting for age, race, education, working hours, and BMI (body mass index).
